# Supplementary material for: The Capacities of the Probiotic Strains L. helveticus MIMLh5 and L. acidophilus NCFM to Induce Th1-Stimulating Cytokines in Dendritic Cells Are Inversely Correlated with the Thickness of Their S-Layers
Source: Biomolecules. 2025 Jul 14;15(7):1012. doi: 10.3390/biom15071012 (PMC12293863; doi:10.3390/biom15071012)
Supplement: Supplementary file 1 [file biomolecules-15-01012-s001.zip › biomolecules-3675899-supplementary.pdf]

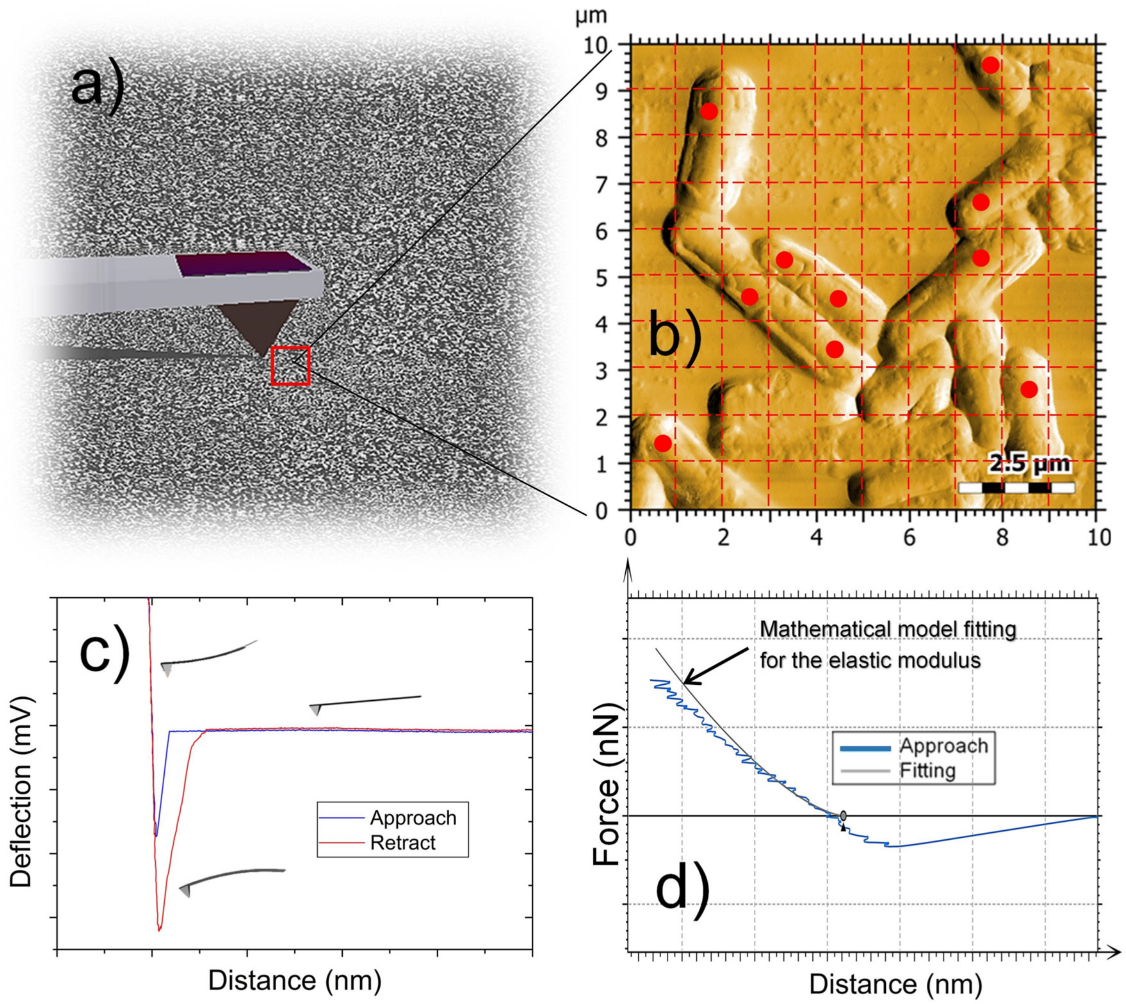

Figure S1: Schematic representation of the 4-step procedure for the AFM analysis of the bacteria surface: scanning of the surface in contact resonance amplitude imaging (CRAI) mode (**a**); creation of the 10-point map for the nanomechanical test (**b**); generation of the force–distance curves (**c**); and fitting procedure for the extrapolation of the elastic modulus (**d**).
